# MagiCMicroRna: a web implementation of AgiMicroRna using shiny

**DOI:** 10.1186/s13029-015-0035-5

**Published:** 2015-03-26

**Authors:** Maarten LJ Coonen, Daniel HJ Theunissen, Jos CS Kleinjans, Danyel GJ Jennen

**Affiliations:** Department of Toxicogenomics, P.O. Box 616, 6200 MD Maastricht, The Netherlands

**Keywords:** Web interface, MicroRNA, Microarray analysis, Data analysis, Agilent

## Abstract

**Background:**

MicroRNA expression can be quantified using sequencing techniques or commercial microRNA-expression arrays. Recently, the *AgiMicroRna* R-package was published that enabled systematic preprocessing and statistical analysis for Agilent microRNA arrays. Here we describe *MagiCMicroRna*, which is a user-friendly web interface for this package, together with a new filtering approach.

**Results:**

We used *MagiCMicroRna* to normalize and filter an Agilent miRNA microarray dataset of cancerous and normal tissues from 14 different patients. With the standard filtering procedure, 250 out of 817 microRNAs remained, whereas the new group-specific filtering approach resulted in broader datasets for further analysis in most groups (>279 microRNAs remaining).

**Conclusions:**

The user-friendly web interface of *MagiCMicroRna* enables researchers to normalize and filter Agilent microarrays by the click of one button. Furthermore, *MagiCMicroRna* provides flexibility in choosing the filtering method. The new group-specific filtering approach lead to an increased number and additional tissue-specific microRNAs remaining for subsequent analysis compared to the standard procedure. The *MagiCMicroRna* web interface and source code can be downloaded from https://bitbucket.org/mutgx/magicmicrorna.git.

**Electronic supplementary material:**

The online version of this article (doi:10.1186/s13029-015-0035-5) contains supplementary material, which is available to authorized users.

## Background

Micro-RNAs (miRNAs) play an important role in post-transcriptional regulation of gene expression by binding to messenger-RNA (mRNA) target genes thereby inducing their silencing. In order to investigate their effect, several commercial array platforms have been developed that quantify the levels of miRNAs present. One of the commonly used miRNA-expression array platforms is manufactured by Agilent. As with all transcriptomics research, data needs to be preprocessed and normalized first before differentially expressed features can be determined. Recently, López-Romero et al. published *AgiMicroRna* [1,2], an integrated analytical R-package enabling systematic preprocessing and statistical analysis for Agilent microRNA arrays. However, *AgiMicroRna* can be too demanding for researchers lacking sufficient programming experience.

Therefore we created *MagiCMicroRna*, a user-friendly web interface for the *AgiMicroRna* package. Besides the new appearance, we have also made some improvements to the original source code. With *MagiCMicroRna*, it is now possible to perform comprehensive preprocessing and filtering of Agilent miRNA arrays by the click of a button.

## Implementation

*MagiCMicroRna* is presented to the user in two ways. General users are invited to use the graphical web-interface that runs locally on their computer using the R-package *shiny* [3]. Three source files need to be downloaded, after which *MagiCMicroRna* can be started using the *runApp* command of shiny. Advanced users that desire to tweak settings or change the analysis according to their specific needs, can also download and edit the source code for running it directly in R.

Both the web interface and source code accept two types of input files. First, the target-file links the raw data files with the experimental design and treatment groups. This file resembles to some extent the original used by López-Romero [1], but contains additional columns in order to perform the group-specific filtering (described in https://bitbucket.org/mutgx/magicmicrorna.git). Secondly, *MagiCMicroRna* needs the raw .txt-files obtained by Agilent Feature Extraction Software.

**Figure 1:**
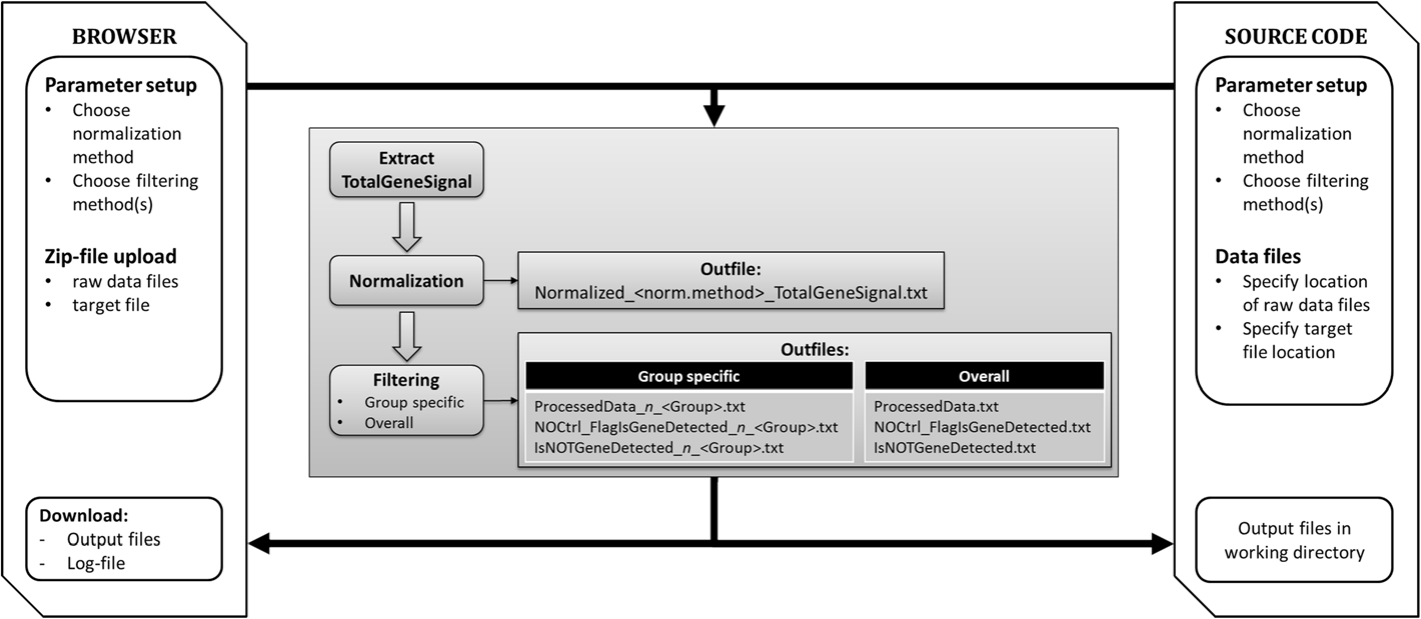
**Overview of the MagiCMicroRna workflow.** Users can either use the web interface (left) or the source code (right).

The *MagiCMicroRna* algorithm is shown in Figure 1. Briefly, it reads the target- and raw datafiles and extracts the summarized TotalGeneSignal for each miRNA. It then normalizes the data based on the algorithm that the user has chosen. Information on the normalization method to use can be found in [2]. The normalized data matrix for all samples is written to the output file *Normalized_<norm.method>_TotalGeneSignal.txt*. In contrast to the original package, the output file *NOCtrl_exprs.txt* is omitted. Instead of normalized data, as was stated by the authors [1], this file contained the raw mean signals for the first occurrence of each miRNA-probe on the array. Subsequently, the user has the option to perform group-specific or overall filtering of miRNAs that passed QC-criteria. Both approaches have their (dis-)advantages. Group-specific filtering applies the filtering criteria to a subset of samples that belong to the respective treatment and control groups. Using this approach prevents interference of aberrant samples on the miRNA-filtering of particular experimental treatments. This is especially useful when the dataset exhibits heterogeneity between groups, where particular miRNAs are only expressed in a small number of samples (e.g. a particular treatment). With overall filtering, these miRNAs would be filtered out before statistical analysis, whilst these can be very interesting to the biologist. Experimental designs that investigate independent treatment effects can greatly benefit from this more directed filtering approach. On the other hand, group-specific filtering creates output files that have different dimensions across experimental groups. In that perspective, overall filtering is recommended for experimental setups where multiple comparisons are of interest, e.g. time series, dose–response or multifactorial designs. In this way the dataset provided to subsequent statistical methods (i.e. linear modeling) does not have missing values. The user thus needs to make a careful decision about the different filtering methods.

In view of the fact that statistical analysis procedures are hard to generalize due to differences in experimental designs, we decided to leave out the linear modelling implemented in the original *AgiMicroRna* package and focus only on the comprehensive normalization and filtering procedures. Our normalized and filtered output files can be used directly in most statistical analysis tools.

## Results and discussion

We used *MagiCMicroRna* to normalize and filter an Agilent miRNA microarray dataset of cancerous and normal tissues from 14 different patients [4] (ArrayExpress E-GEOD-14985). Both group-specific and overall filtering approaches were applied and the results are shown in Table 1. With the overall filtering procedure, a total of 250 out of 817 miRNAs can be used for subsequent analyses. The dataset used here is derived from independent sources (different tissues from different subjects). Therefore, preselecting samples belonging to the same experimental group (tissue) before filtering (group-specific filtering) can be extremely useful. Compared to the overall filtering procedure, the total number of miRNAs remaining with group-specific filtering was higher in all groups, except for Liver_Tumor (232 miRNAs remain). Furthermore, some tissue-specific miRNAs were found only with group-specific filtering (Additional file 1), such as hsa-miR-122 (liver-injury) [5], hsa-miR-125b* (breast cancer) [6] and hsa-miR-371a-3p (testes tumor) [7].

**Table 1:** **Group-specific filtering increases the number of filtered miRNAs that remain for subsequent (statistical) analyses**

**Experimental group**	**miRNAs filtered**	
	**Group-specific**	**Overall**
Breast_Tumor	336	250
Colon_Tumor	280	
Liver_Tumor	232	
Lung_Tumor	334	
Lymphoma_Tumor	314	
Ovary_Tumor	279	
Prostate_Tumor	300	
Testes_Tumor	405	

These findings are clearly demonstrating the added value of group-specific filtering in case of heterogeneous datasets where multiple comparisons are not required.

## Conclusion

*MagiCMicroRna* continues on the foundation established by the original *AgiMicroRna* package. It is presented to the user as executable source code or as a user friendly web interface, enabling researchers without sufficient programming experience to normalize and filter their Agilent miRNA data. Besides the new appearance, some parts of the original source code have been modified to generate a normalized data file and perform a new filtering procedure, which enables group-specific filtering of miRNAs. Tested on a publicly available miRNA-dataset, *MagiCMicroRna* provided more flexibility in choosing the filtering method, in this case leading to an increased amount and additional tissue-specific miRNAs compared to the standard procedure.

## Availability and requirements

- **Project name:***MagiCMicroRna*
- **Project home page:**https://bitbucket.org/mutgx/magicmicrorna.git
- **Operating system(s**): Platform independent
- **Programming language:** R/shiny
- **Other requirements:** R packages *AgiMicroRna, shiny* and *shinyIncubator*
- **License:** GPLv3
- **Any restrictions to use by non-academics:** none

## Additional file

Additional file 1:
**microRNAs present in all filtering approaches.** Unique microRNAs for each group-specific filtering approach.
