# Supplementary material for: MagiCMicroRna: a web implementation of AgiMicroRna using shiny
Source: Source Code Biol Med. 2015 Mar 26;10:4. doi: 10.1186/s13029-015-0035-5 (PMC4383057; doi:10.1186/s13029-015-0035-5)
Supplement: Additional file 1: — microRNAs present in all filtering approaches. Unique microRNAs for each group-specific filtering approach. [file 13029_2015_35_MOESM1_ESM.pdf]

microRNAs present in all filtering approaches

ebv-miR-BART13	hsa-miR-146b-5p	hsa-miR-20b	hsa-miR-342-5p	hsa-miR-572
hcmv-miR-UL70-3p	hsa-miR-148a	hsa-miR-21	hsa-miR-34a	hsa-miR-574-3p
hsa-let-7a	hsa-miR-148b	hsa-miR-21*	hsa-miR-34b*	hsa-miR-574-5p
hsa-let-7b	hsa-miR-150	hsa-miR-210	hsa-miR-361-3p	hsa-miR-575
hsa-let-7c	hsa-miR-150*	hsa-miR-214	hsa-miR-361-5p	hsa-miR-590-5p
hsa-let-7d	hsa-miR-151-3p	hsa-miR-214*	hsa-miR-362-5p	hsa-miR-625
hsa-let-7e	hsa-miR-151-5p	hsa-miR-22	hsa-miR-363	hsa-miR-630
hsa-let-7f	hsa-miR-152	hsa-miR-221	hsa-miR-365	hsa-miR-638
hsa-let-7g	hsa-miR-154	hsa-miR-221*	hsa-miR-374a	hsa-miR-652
hsa-let-7i	hsa-miR-155	hsa-miR-222	hsa-miR-374b	hsa-miR-654-3p
hsa-miR-100	hsa-miR-15a	hsa-miR-223	hsa-miR-376a	hsa-miR-660
hsa-miR-101	hsa-miR-15b	hsa-miR-224	hsa-miR-376c	hsa-miR-663
hsa-miR-103	hsa-miR-16	hsa-miR-23a	hsa-miR-377	hsa-miR-768-3p
hsa-miR-106a	hsa-miR-17	hsa-miR-23b	hsa-miR-378	hsa-miR-768-5p
hsa-miR-106b	hsa-miR-17*	hsa-miR-24	hsa-miR-378*	hsa-miR-769-5p
hsa-miR-107	hsa-miR-181a	hsa-miR-25	hsa-miR-379	hsa-miR-874
hsa-miR-10a	hsa-miR-181b	hsa-miR-26a	hsa-miR-381	hsa-miR-886-3p
hsa-miR-10b	hsa-miR-181c	hsa-miR-26b	hsa-miR-382	hsa-miR-923
hsa-miR-1224-5p	hsa-miR-181d	hsa-miR-27a	hsa-miR-409-3p	hsa-miR-92a
hsa-miR-1225-5p	hsa-miR-183	hsa-miR-27b	hsa-miR-423-5p	hsa-miR-93
hsa-miR-1228	hsa-miR-185	hsa-miR-28-5p	hsa-miR-424	hsa-miR-939
hsa-miR-125a-3p	hsa-miR-186	hsa-miR-299-5p	hsa-miR-425	hsa-miR-940
hsa-miR-125a-5p	hsa-miR-188-5p	hsa-miR-29a	hsa-miR-451	hsa-miR-95
hsa-miR-125b	hsa-miR-18a	hsa-miR-29b	hsa-miR-454	hsa-miR-96
hsa-miR-126	hsa-miR-18b	hsa-miR-29b-1*	hsa-miR-455-3p	hsa-miR-98
hsa-miR-127-3p	hsa-miR-192	hsa-miR-29c	hsa-miR-455-5p	hsa-miR-99a
hsa-miR-128	hsa-miR-193a-3p	hsa-miR-29c*	hsa-miR-483-5p	hsa-miR-99b
hsa-miR-130a	hsa-miR-193a-5p	hsa-miR-301a	hsa-miR-484	hsv1-miR-LAT
hsa-miR-130b	hsa-miR-193b	hsa-miR-30a	hsa-miR-486-5p	kshv-miR-K12-3
hsa-miR-132	hsa-miR-194	hsa-miR-30a*	hsa-miR-487b	
hsa-miR-133b	hsa-miR-195	hsa-miR-30b	hsa-miR-493*	
hsa-miR-134	hsa-miR-196b	hsa-miR-30b*	hsa-miR-494	
hsa-miR-135a*	hsa-miR-197	hsa-miR-30c	hsa-miR-495	
hsa-miR-139-3p	hsa-miR-199a-5p	hsa-miR-30c-1*	hsa-miR-497	
hsa-miR-139-5p	hsa-miR-199b-3p	hsa-miR-30d	hsa-miR-500*	
hsa-miR-140-3p	hsa-miR-199b-5p	hsa-miR-30e	hsa-miR-501-3p	
hsa-miR-140-5p	hsa-miR-19a	hsa-miR-30e*	hsa-miR-501-5p	
hsa-miR-142-3p	hsa-miR-19b	hsa-miR-320	hsa-miR-502-3p	
hsa-miR-143	hsa-miR-19b-1*	hsa-miR-324-3p	hsa-miR-502-5p	
hsa-miR-145	hsa-miR-200b	hsa-miR-324-5p	hsa-miR-505	
hsa-miR-145*	hsa-miR-200c	hsa-miR-331-3p	hsa-miR-505*	
hsa-miR-146a	hsa-miR-204	hsa-miR-340	hsa-miR-532-3p	
	hsa-miR-20a	hsa-miR-342-3p	hsa-miR-532-5p	

Unique microRNAs for each group-specific filtering approach

Breast	Testes	Lung	Lymphoma	Colon	Liver	Ovary	Prostate
ebv-miR-BART4	hsa-miR-105*	hsa-let-7a*	hsa-miR-155*	hsa-miR-147b	hsa-miR-122	hsa-miR-383	ebv-miR-BART19-3p
hsa-miR-1228*	hsa-miR-182*	hsa-miR-138	hsa-miR-877*	hsa-miR-490-3p	hsa-miR-122*	hsa-miR-449b	hsa-miR-106a*
hsa-miR-125b-1*	hsa-miR-188-3p	hsa-miR-15b*		hsa-miR-552	hsa-miR-885-3p	hsa-miR-506	hsa-miR-206
hsa-miR-129-5p	hsa-miR-200c*	hsa-miR-29a*		hsa-miR-592	hsa-miR-885-5p	hsa-miR-507	hsa-miR-491-5p
hsa-miR-23b~*	hsa-miR-302a	hsa-miR-545				hsa-miR-508-3p	hsa-miR-582-3p
hsa-miR-26a-2*	hsa-miR-302a*					hsa-miR-508-5p	hsa-miR-933
hsa-miR-335*	hsa-miR-302b					hsa-miR-509-3-5p	
hsa-miR-33b*	hsa-miR-302b*					hsa-miR-509-3p	
hsa-miR-422a	hsa-miR-302c					hsa-miR-510	
hsa-miR-492	hsa-miR-302d						
hsa-miR-548c-3p	hsa-miR-329						
hsa-miR-550	hsa-miR-337-3p						
hsa-miR-566	hsa-miR-367						
hsa-miR-610	hsa-miR-371-3p						
hsa-miR-645	hsa-miR-372						
hsa-miR-648	hsa-miR-373						
hsa-miR-659	hsa-miR-377*						
hsa-miR-769-3p	hsa-miR-433						
hsa-miR-934	hsa-miR-512-3p						
hsa-miR-936	hsa-miR-512-5p						
	hsa-miR-515-3p						
	hsa-miR-515-5p						
	hsa-miR-516b						
	hsa-miR-517b						
	hsa-miR-518a-3p						
	hsa-miR-518b						
	hsa-miR-518c						
	hsa-miR-518d-5p						
	hsa-miR-518e	Testes (cont'd.)					
	hsa-miR-518e*	hsa-miR-520g					
	hsa-miR-518f	hsa-miR-520h					
	hsa-miR-518f*	hsa-miR-523					
	hsa-miR-519b-3p	hsa-miR-524-3p					
	hsa-miR-519c-3p	hsa-miR-524-5p					
	hsa-miR-519d	hsa-miR-525-5p					
	hsa-miR-519e	hsa-miR-615-3p					
	hsa-miR-519e*	hsa-miR-628-3p					
	hsa-miR-520a-3p	hsa-miR-767-5p					
	hsa-miR-520a-5p	hsa-miR-888					
	hsa-miR-520b	hsa-miR-890					
	hsa-miR-520c-3p	hsa-miR-891a					
	hsa-miR-520d-3p	hsa-miR-891b					
	hsa-miR-520f	hsa-miR-892a					
